# Editorial: Tumor adaptation to cellular stresses: mechanisms, biomarkers and therapeutic opportunities

**DOI:** 10.3389/fmed.2023.1268976

**Published:** 2023-08-17

**Authors:** Michela Cortesi, Giacomo Rossino, Anindita Chakrabarty, Daniela Rossi

**Affiliations:** ^1^Biosciences Laboratory, IRCCS Istituto Romagnolo per lo Studio dei Tumori (IRST) “Dino Amadori”, Meldola, Italy; ^2^Department of Drug Sciences, University of Pavia, Pavia, Italy; ^3^Department of Life Sciences, Shiv Nadar Institution of Eminence, Greater Noida, Uttar Pradesh, India

**Keywords:** metabolic stress, oxidative stress, ROS, hypoxia, mitochondrial stress, ER stress, tumor adaptation

One of the major challenges of cancer treatment is cancer cells' ability to adapt and survive under stressful conditions. To overcome physiological and microenvironmental stresses (i.e., oxidative stress, hypoxia, nutrient deprivation, endoplasmic reticulum and mitochondrial stresses, and DNA damage), tumor cells reprogram their signaling pathways in a spatial and temporal manner through the alteration of transcriptional, translational, and post-translational machinery. This results in the clonal expansion of tumor cells with distinct gene expression and metabolic signatures. Tumors' phenotypic heterogeneity, induced as a result of the stress response, drives therapy resistance, tumor relapse, and metastasis.

Different biological mechanisms underpin cancer cells' adaptation to stressful conditions. For instance, damage-induced senescence may activate the p16 or the p53–p21 axis, leading to altered cell proliferation and metabolic reprogramming affecting tumor progression, ECM plasticity, and vascular permeability (1, 2). Anticancer drugs can prompt the accumulation of misfolded proteins and induce ER stress, leading to reactive oxygen species (ROS) generation (3, 4). In the presence of high levels of ROS, tumor cells can alter sulfur-based metabolism, NADPH generation, and the activity of antioxidant transcription factors (5). Under hypoxic conditions, the induction of hypoxia-inducible factor 1 alpha (HIF-1α) causes the upregulation of the glycolytic metabolic pathway, enhancing adenosine triphosphate (ATP) production required for cell survival and proliferation (6). Endogenous and exogenous stresses may lead to the production of a plethora of molecules with either beneficial or harmful roles in tumor progression and therapy response. However, our understanding of how cancer cells manage these stresses is far from complete.

The aim of this Research Topic is to highlight recent advances in the research on mechanisms of tumor cells' adaptation to stressful conditions, as well as the biomarkers thereof and the possible therapeutic implications.

Protein synthesis, consuming almost 1/3 of the total cellular energy, is one of the first processes to be inhibited as an adaptive stress response (7). The shutdown of global mRNA translation under diverse stresses happens through three major pathways. The Integrated Stress Response (ISR), Unfolded Protein Response (UPR), and mammalian Target of Rapamycin Complex 1 (mTORC1) pathways inhibit initiation and the AMPK-eEF2K axis inhibits elongation (8).

Warrier et al. highlight a diminished, albeit continuous, mRNA translation under matrix-deprivation stress in breast cancer cells. Their results reveal a concurrent inhibition of translation initiation (eIF2α) and elongation (eEF2) factors and of the mTORC1 pathway in response to matrix deprivation. The authors suggest an altered translatome, exploitable by these cells to survive under matrix-deprivation conditions. Moreover, integrated omics analysis reveals the enrichment of candidate proteins with roles in cancer progression and/or stress response, among which the Nucleic acid Binding Protein (CNBP) seems an important player in stress adaptation. These data point to matrix deprivation as an adaptive strategy during metastasis leading to the reprogramming of protein synthesis.

As also reported by Mijit et al., ISR is critical for the adaptation and survival of cells under environmental stresses. These authors investigate the role of ISR following Ref-1 redox signaling inhibition in both PDAC cells and cancer-associated fibroblasts (CAFs) and in a co-culture model of these cell types. Their data highlight Ref-1 inhibition as a trigger for ISR activation mainly through the Ref-1/eIF2/ATF4 axis, modulated by PERK kinase. Additionally, they show that Ref-1 and PERK concomitant inhibition activates the ISR pathway through general control non-depressible 2 kinase (GCN2), contributing to enhanced cell killing as compared to ISR activation via PERK signaling. Notably, the effects of PERK or GCN2 inhibition are minimal on both tumor and CAFs in monolayer as compared to 3D co-cultures, underlying the importance of using complex *in vitro* models to study cellular stress response.

One important hallmark of cancer is metabolic reprogramming. Recently, lysosomes have emerged as master regulators of cellular nutrient sensing and adaptation to metabolic stresses. Lengauer et al. show that glutamine starvation induced LC3-dependent autophagy in cancer cells, which is prevented by V-ATPase function inhibition. However, the combination of glutamine deprivation with the administration of the anticancer agent archazolid (lysosomal V-ATPase inhibitor) does not lead to therapeutic benefit. Cells circumvent cell death and growth inhibition by increased glutamine uptake, augmented lactate production, and increased hexokinase activity. The deepening of the underpinning mechanisms shows that glutamine starvation leads to lysosomal acidification together with an elevated expression and activity of amino acid transporters SLC1A5, SLC38A1. These authors conclude that metabolic plasticity likely contributes to the failure of glutamine deprivation combined with archazolid also increasing the survival capabilities of tumor cells under stressful conditions.

The heterogeneity of tumor cells and their microenvironment plays a central role in stress response, fuelling disease progression, and treatment resistance (9). Liu et al., using 452 colon adenocarcinoma (COAD) samples retrieved from The Cancer Genome Atlas (TCGA), define a novel intratumor heterogeneity (ITH)-related prognostic signature to predict overall survival (OS) of patients. The signature consists of five tumor-related genes, CEACAM5, ENO2, GABBR1, SLC44A4, and MC1R, which results in an independent prognostic factor for predicting the 1, 3, and 5-year OS of COAD patients. The signature shows potential implications in treatment decision-making, deciphering the role of the tumor microenvironment (TME) especially in the context of immune cell infiltration and chemotherapy response. These findings warrant further investigations to validate the significance of this signature.

The Research Topic closes with a review article on the therapeutic effects of cold atmospheric plasma (CAP) on solid tumors by Min et al..

CAP is a partially ionized gas that induces reactive oxygen and nitrogen species production which, together with the oxidative stress responses that they trigger, have shown the ability to selectively kill tumor cells. In their publication, the authors introduce currently available CAP devices and treatment methods while also discussing the CAP components responsible for the antitumor activity along with their mechanism of action. They emphasize studying the selectivity of CAP toward malignant neoplasms by analyzing its effects on tumor cells and on the TME.

The five articles in this Research Topic demonstrate that cancer cells exhibit very distinctive metabolism, genetic expression, and signaling pathways to adapt to microenvironmental and therapeutic stresses. A clear understanding of these adaptive mechanisms will enable us to develop and deliver safer and more effective anticancer therapeutic strategies.

## Author contributions

MC: Conceptualization, Writing—original draft, Writing—review and editing. GR: Conceptualization, Writing—original draft, Writing—review and editing. AC: Supervision, Writing—review and editing. DR: Conceptualization, Supervision, Writing—review and editing.
